# Antiobesity Medications for Older Adults—the New, the Good, the Bad, and the Unknown

**DOI:** 10.1002/oby.70029

**Published:** 2025-10-08

**Authors:** Anna Pendrey, Javier Sevilla‐Martir

**Affiliations:** ^1^ Department of Family Medicine Indiana University School of Medicine Indianapolis Indiana USA

**Keywords:** antiobesity medications, obesity, older adults

## Abstract

**Objective:**

Obesity affects 42% of older adults, with rates continuing to rise. This a complex condition influenced by non‐modifiable as well as modifiable risk factors. The disease can be treated through modifications to diet, physical activity, and behavior and more recently through antiobesity medications (AOMs) and surgery. Treatment must be tailored to individual needs due to age‐related metabolic and physiological changes. This review aimed to identify the suitability of seven FDA‐approved AOMs in the treatment of obesity in older adults.

**Methods:**

A review of AOMs was performed, focusing on their efficacy in weight loss, side effects, and potential health risks in older adults. Studies were selected to later evaluate the overall suitability and safety of these medications.

**Results:**

AOMs can improve cardiovascular outcomes, hypertension, hyperlipidemia, metabolic liver disease, obstructive sleep apnea, and chronic kidney disease. Weight loss in older adults using AOMs is associated with an increased risk of sarcopenia.

**Conclusions:**

From a policy standpoint, ensuring coverage of AOMs for older adults is critical, as these medications help reduce obesity‐related complications. However, increased participation in clinical trials is urgently needed to study the impact of quality of life and outcomes in older adults.

## 1 Introduction—The Complex Landscape of Obesity in Older Adults

Obesity is a chronic disease with a relapsing and progressive process. Its prevalence was 42% in 2020 [1, 2, 3, 4, 5]. Obesity in older adults is a complex condition influenced by non‐modifiable as well as modifiable risk factors. The disease can be treated through modifications to diet, physical activity, and behavior and more recently through antiobesity medications (AOMs) and surgery [6]. In this article, we will focus on Food and Drug Administration (FDA)‐approved medications for weight loss in older adults [6]. Older adults are considered those 65 years of age and older. See the classification of obesity in Table 1.

**TABLE 1:** BMI classification based on WHO.

Underweight	Normal weight	Overweight	Obesity	Obesity I, mild risk	Obesity II, moderate risk	Obesity III, severe risk	Obesity IV, severe risk	Obesity V, severe risk
BMI < 18.4 kg/m^2^	BMI 18.5–24.9 kg/m^2^	BMI 25–29.9kg/m^2^	BMI > 30 kg/m^2^	BMI > 30–34.9 kg/m^2^	BMI > 35–39.9 kg/m^2^	BMI > 40–49.9 kg/m^2^	BMI > 50–59.9 kg/m^2^	BMI> 60 kg/m^2^

*Note*: Body mass index (BMI): weight in kilograms divided by height in meters squared (kg/m^2^).

Abbreviation: WHO, World Health Organization.

## 2 Approach to Using Medications in Older Adults With Obesity

We recommend that the initial management of obesity in older adults should include intensive lifestyle interventions that combine diet, exercise, and behavioral modifications [7, 8, 9]. The primary objective in managing an older adult with obesity is to identify the individual's treatment goals, which aim to improve obesity‐related complications and enhance quality of life. A secondary objective is to identify older adults who are candidates for safe and effective weight loss. According to the American Association of Clinical Endocrinology (AACE) guidelines, lifestyle changes, including improving diet and increasing physical activity, are recommended for individuals with a body mass index (BMI) between 25 kg/m^2^ and 29.9 kg/m^2^ and without any weight‐related complications [6]. AOMs are indicated in older adults with BMI > 30 kg/m^2^ or 27 kg/m^2^ with one or more obesity‐related comorbidities and who have an inadequate response to lifestyle interventions alone. The decision to initiate AOMs in older adults should be individualized and tailored to the older adults' potential risks and benefits, functional status, comorbid conditions, preferences, and goals of care [9, 10]. See treatment guideline according to BMI in Table 2. The potential impact of weight loss on physical function, muscle loss, bone loss, and overall health status should guide treatment decisions regarding the use of AOMs in older adults with obesity.

**TABLE 2:** Treatment guideline based on BMI.

BMI ≥ 25 kg/m^2^	BMI ≥ 27 kg/m^2^ with 1 or more comorbidities	BMI ≥ 30 kg/m^2^	BMI ≥ 35 kg/m^2^ with comorbidity	BMI > 40 kg/m^2^
	Pharmacotherapy	Pharmacotherapy	Pharmacotherapy	Pharmacotherapy
			Consider Bariatric Surgery	Bariatric Surgery
Diet, Exercise, and Behavioral Modifications	Diet, Exercise, and Behavioral Modifications	Diet, Exercise, and Behavioral Modifications	Diet, Exercise, and Behavioral Modifications	Diet, Exercise, and Behavioral Modifications

AOMs should be continued if a patient has lost at least 5% of body weight after 3 months of therapy. If this is not achieved or the patient has intolerable side effects to the medication, discontinuation of the medication is recommended; an alternative medication or treatment method should be sought [11]. Weight loss of 10% can lead to significant clinical improvement in several medical conditions in older adults, including type 2 diabetes mellitus (T2DM), hypertension, and cardiovascular disease [11, 12, 13, 14, 15]. It is important to start AOMs with the lowest dose and increase the dose as tolerated and according to guidelines. In this review, we outline all the FDA‐approved medications and briefly describe their mechanism of action, adverse effects (AEs), and specific caveats in older adults.

## 3 Pharmacological Management for Obesity

We will review seven FDA‐approved AOMs and their suitability for older adults in the treatment of obesity. Phentermine is approved for short‐term weight management. The other AOMs have been approved for long‐term weight management. These include phentermine and topiramate extended‐release, naltrexone and bupropion sustained‐release (centrally acting), orlistat (peripherally acting), and liraglutide, semaglutide, and tirzepatide (centrally and peripherally acting) [11, 13, 14].

### 3.1 Phentermine (Adipex‐P, Lomaira)

This medication is a sympathomimetic agent. It acts in the hypothalamus, increasing norepinephrine release and activating adrenergic receptors to suppress appetite and decrease food consumption. Phentermine can be used for up to 12 weeks, although clinicians may prescribe it for longer periods due to the nature of obesity as a chronic disease [16]. A 36‐week randomized controlled trial (RCT) of phentermine studied 64 patients who were assigned to three arms: placebo, phentermine 30 mg daily, or intermittent phentermine 30 mg daily consisting of a 4‐week on and 4‐week off prescription pattern. Both phentermine groups lost ~13 kg of their initial weight, while the placebo group lost only 4.8 kg [14, 16]. Notably, this study did not include older adults [16].

For older adults, we recommend prescribing phentermine with great caution, as this medication can cause dizziness and may lead to an increased risk of falls [17]. Other side effects include headache, palpitations, insomnia, nausea, constipation, and dysgeusia, all of which are commonly observed in older adults. This medication can worsen anxiety, a disorder that affects 11.2% of older adults [18]. AEs include exacerbation of glaucoma, hyperthyroidism, coronary artery disease, and myocardial infarction due to its sympathomimetic properties. In older adults with hypertension, the medication should be used with close monitoring. Alcohol consumption while taking this medication is not recommended. In our practice, we generally avoid this medication for older adults, who may have a multitude of comorbidities. Serious side effects with phentermine include primary pulmonary hypertension and hypoglycemia when taken with antidiabetic medications.

Phentermine is contraindicated for older adults who have coronary artery disease or uncontrolled hypertension, glaucoma, or overactive thyroid disease. In addition, we avoid phentermine in older adults who were taking monoamine oxidase inhibitors (MAOIs) within the past 14 days. Avoid phentermine in older adults with a history of drug abuse, as phentermine is a controlled medication that can lead to abuse and dependence.

### 3.2 Phentermine/Topiramate Extended‐Release ER (Qsymia)

Approved in 2012, phentermine and topiramate, a neuro‐stabilizer gamma‐aminobutyric acid (GABA) modulator, carbonic anhydrase inhibitor, and dopamine/noradrenaline reuptake inhibitor, can induce weight loss up to 10.2% at a high dose at 1 year [19]. Its efficacy was demonstrated in the CONQUER study where patients with overweight or obesity were randomized to phentermine/topiramate 7.5 mg/46 mg (low‐dose), 15 mg/92 mg (high‐dose), or placebo [19, 20]. The mean age of participants was 51.1 years. The baseline BMI range was 36.2–36.7 kg/m^2^. At week 56, weight loss of 8.6% was achieved in the high‐dose group, 6.6% in the low‐dose group, and 1.2% in the placebo group [20]. The SEQUEL trial, a 2‐year extension of CONQUER, noted continued weight loss of 9.3% with low dose and 10.5% with a high dose [21]. The EQUIP study randomized participants 18–70 years old with BMI > 35 kg/m^2^ to phentermine/topiramate 3.75/23 mg, 15/92 mg, and placebo, in addition to lifestyle interventions, for 56 weeks. The mean body weight loss for the placebo, low‐dose, and high‐dose groups was 1.6%, 5.1%, and 10.9%, respectively. The high‐dose group relative to placebo demonstrated improved systolic and diastolic blood pressure, triglyceride levels, total cholesterol, high‐density lipoprotein (HDL), low‐density lipoprotein (LDL), and fasting glucose [22, 23]. Only 7% of the participants in these three studies for phentermine/topiramate were older adults [19, 23].

The most common side effects of phentermine/topiramate include dizziness, lightheadedness, constipation, and dry mouth. Contraindications are the same as listed for phentermine.

An additional contraindication is its use in older adults with mood disorders, as suicidal thoughts and/or actions may occur. Like phentermine, phentermine/topiramate is a controlled substance. We generally use this medication cautiously or fully avoid it when prescribed in combination with diuretics, such as hydrochlorothiazide (hypokalemia), carbonic anhydrase inhibitors, such as acetazolamide (nephrolithiasis and metabolic acidosis), and antiseizure medications, such as valproic acid (hyperammonemia). A dose higher than 7.5 mg/46 mg should not be prescribed in older adults with liver or kidney disease.

It is recommended to start phentermine/topiramate at a dose of 3.75 mg/23 mg daily for the first 12 weeks. If there is < 3% weight loss at the end of 12 weeks, we consider stopping the medication or increasing its dose. For the next 12 weeks, the dose can be increased to 7.5 mg/46 mg daily. If the target weight loss has not been reached after this time, the dose can be increased to 11.25 mg/69 mg for an additional 2 weeks. If the medication is tolerated well, the dose may ultimately be increased to 15 mg/92 mg. To discontinue phentermine/topiramate in older adults, it is advised to taper off the medication instead of abruptly stopping phentermine/topiramate.

### 3.3 Naltrexone/Bupropion Extended‐Release ER (Contrave)

Approved in 2014, bupropion is a dopamine and noradrenaline reuptake inhibitor, and naltrexone is a μ‐opioid receptor antagonist. Naltrexone/bupropion ER acts in the hypothalamic and mesolimbic dopamine circuits to promote satiety, reduce food intake, and increase energy expenditure, causing weight loss. The CONTRAVE Obesity Research‐1 (COR‐1) RCT involved participants with BMI of 30–45 kg/m^2^ with uncomplicated obesity, or BMI 27–45 kg/m^2^ with controlled hypertension and dyslipidemia. Participants were randomized to receive naltrexone/bupropion ER 32 mg/360 mg, naltrexone/bupropion ER 16 mg/360 mg, or placebo. At week 56, weight loss achieved from baseline was 6.1%, 5.0%, and 1.3%, respectively [23, 24, 25]. The trial did not evaluate the effect of this drug on the mortality and quality of life of the participants [24]. Only 2% of the COR trial participants were older adults [23].

Side effects include nausea, headache, and anxiety. Naltrexone/bupropion ER can cause central AEs, such as dizziness; therefore, it should be used with caution in older adults [25]. To minimize side effects, the dose should be titrated gradually. As with phentermine, the use of this medication is not advised in older adults with uncontrolled hypertension, eating disorders, seizure disorders, and alcohol withdrawal [25].

Naltrexone/bupropion ER 8 mg/90 mg should be titrated as follows in older adults: week 1, one tablet in the morning; week 2, increase the dose to one tablet in the morning and one in the evening. If tolerated well at week 3, the dose can be increased to two tablets in the morning and one in the evening. In week 4, two tablets in the morning and two in the evening can be prescribed. Its maximum dose is 32 mg/360 mg daily. Special consideration must be taken in older adults with the following conditions: kidney impairment, where the dose needs to be reduced to one tablet twice daily; and moderate to severe hepatic impairment, where the dose should be reduced to one tablet daily. Importantly, there is limited data on naltrexone/bupropion in older adults over 65 years and no data in those 75 years and older. Naltrexone and bupropion are both metabolized by the kidneys, and more side effects could be seen in older adults as renal function is reduced.

### 3.4 Orlistat (Xenical and aLLi)

This medication was approved in 1999 and introduced as an over‐the‐counter (OTC) medication in 2007. Orlistat reduces fat absorption by inhibiting gastrointestinal (GI) and pancreatic lipases [26]. In a prospective pilot study of 13 older adult women with obesity prescribed orlistat for 6 months, total weight loss was 9.4% [23]. The Xenical in the Prevention of Diabetes in Obese Subjects Study (XENDOS), where older adults were not included, involved participants with obesity but without diabetes. Adults with a mean age of 43 and a baseline BMI of 37 kg/m^2^ were randomized to 120 mg three times daily of orlistat vs. placebo for 4 years. Mean weight loss with orlistat was 10.6 kg after 1 year vs. 6.2 kg with placebo. At 4 years, weight loss compared to baseline was 5.8 kg vs. 3.0 kg, with 52.8% of orlistat and 37.3% of placebo participants losing at least 5% of their body weight [27]. Short follow‐up duration, including only patients with obesity with glucose intolerance, and lack of cardiovascular outcome assessment limited this trial's generalizability and outcome interpretation [11]. Treatment with orlistat 120 mg is associated with considerable improvements in cardiovascular risk factors, which include a decrease in LDL cholesterol, fasting glucose, and systolic and diastolic blood pressure after 1 year of treatment [26].

**TABLE 3:** Medications approved by the Food and Drug Administration for treatment for obesity and use for older adults.

Medications	Phentermine (Adipex‐P, Lomaira)	Orlistat (aLLi, Xenical)	Liraglutide (Saxenda)	Phentermine/Topiramate (Qsymia)	Naltrexone‐Bupropion (Contrave)	Semaglutide (Wegovy)	Tirzepatide (Zepbound)
Expected weight loss	5%–10%	5%–10%	5%–10%	5%–10%	5%–10%	10%–20%	> 15%–20%
Mechanism of action	Norepinephrine releasing agent that suppresses appetite	Pancreatic and gastric lipase inhibitor; resulting in fat malabsorption; reduces net energy intake	GLP‐1 agonist delays gastric emptying to suppress appetite and reduce food intake	Phentermine is a norepinephrine releasing agent suppressing appetite; topiramate is a GABA receptor modulator that reduces food intake	Naltrexone is an opioid antagonist and dopamine and norepinephrine reuptake inhibitor; bupropion acts on CNS pathways to reduce food intake	GLP‐1 agonist delays gastric emptying to suppress appetite and reduce food intake	GLP‐1 agonist delays gastric emptying to suppress appetite and reduce food intake, decrease insulin secretion; GIP decrease insulin secretion, increase glucagon, increase adipocyte metabolism
Weight loss expected in older adults with treatment	No studies reported in older adults	5%–10%, 52 weeks [19, 23]	8%, 56 weeks [19, 23]	5%–10.2%, 52 weeks [19, 23]	5%–10%, 56 weeks [19, 23]	14.8%, 68 weeks [19, 23]	25.3%, 88 weeks [19, 23]
Available forms	Adipex‐P 37.5 mg and Lomaira 8 mg tab, capsules 15 mg, 30 mg, 37.5 mg	aLLi 60 mg over the counter, Xenical 120 mg prescribed	0.6 mg, 1.2 mg, 1.8 mg, 2.4 mg, 3.0 mg	Phentermine/Topiramate Extended‐Release (ER) Cap 3.75 mg /23 mg, 7.5 mg/46 mg, 11.5 mg/69 mg, 15 mg/92 mg	Naltrexone/Bupropion Extended‐Release (ER) Tab 8 mg/90 mg	0.25 mg, 0.5 mg, 1.0 mg, 1.7 mg, 2.4 mg SC injection	2.5 mg, 5 mg, 7.5 mg, 10 mg, 12.5 mg, 15 mg SC injection
Dose	Short‐term treatment up to 12 weeks; Can give daily, BID; Max 37.5 mg/day; Give 1–2 hours after breakfast	Long‐term treatment > 12 weeks; aLLi 60 mg before meals PO TID; Xenical 120 mg before meals PO TID	Long‐term treatment > 12 weeks; Starting dose 0.6 mg SC daily; Dose increased weekly by 0.6 mg SC as tolerated to reach 3.0 mg SC daily	Long‐term treatment > 12 weeks; Starting dose 3.75 mg/23 mg ER Q am for 14 days; Then increase to 7.5 mg/46 mg Q am; Max 15 mg/92 mg; Weight loss < 3% after 12 weeks in 7.5 mg/46 mg increase to 11.5 mg/69 mg Q am for 14 days; And then to 15 mg/92 mg daily; Discontinue if weight loss < 5% in 12 weeks and taper medication to take 15 mg/92 mg every other day for at least 1 week to discontinue	Long‐term treatment > 12 weeks; Starting dose 8 mg of naltrexone and 90 mg of bupropion daily for 1 week; Those subsequently increase each week by 1 tablet per day until maintenance dose of 2 tablets twice a day at week 4; Max 4 tablets/day; Discontinue if weight loss < 5% after 12 weeks on max dose; Do not cut/crush/chew tablet; avoid administration with high‐fat meal	Long‐term treatment > 12 weeks; Starting dose 0.25 mg SC weekly for 4 weeks; Increase to 0.5 mg SC weekly for 4 weeks, then to 1 mg SC weekly for 4 weeks, then to 1.7 mg SC weekly for 4 weeks; Max dose 2.4 mg SC weekly	Long‐term treatment > 12 weeks ; Starting dose 2.5 mg SC weekly; Increase dose every 4 weeks by 2.5 mg as tolerated; Max dose 15 mg SC weekly
Weight loss relative to placebo and treatment dose and duration	30 mg: 12.2 kg Placebo: 4.8 kg 36 weeks [16]	120 mg: 10.6 kg Placebo: 6.2 kg 52 weeks [27] 120 mg: 5.8 kg Placebo: 3.0 kg 48 months [27]	3.0 mg: 8.4 kg Placebo: 2.8 kg 56 weeks [34] 3.0 mg: 6.0% 1.8 mg: 4.7% Placebo: 4.7% 56 weeks [35]	15/92 mg: 10.9% 3.75/23 mg: 5.1% Placebo: 1.6% 56 weeks [22, 23]	32 mg/360 mg: 6.1% 16 mg/360 mg: 5.0% Placebo: 1.3% 56 weeks [23, 24, 25]	2.4 mg: 14.9% Placebo: 2.4% 68 weeks [37] 2.4 mg: 9.6 % Placebo: 3.4% 68 weeks [38]	15 mg: 20.9% 10 mg: 19.5% 5 mg: 15.0% Placebo: 3.1% 72 weeks [50] 15 mg: 14.7% 10 mg: 12.8% Placebo: 3.2% 72 weeks [51]
Side effects	Dependency, angina, myocardial infarction, hypertension	Flatus with discharge, fecal urgency, oily evacuations, increased defecation, fecal incontinence	Nausea, vomiting, constipation, diarrhea, headache, fatigue, dizziness, abdominal pain, increased lipase levels	Insomnia, dry mouth, constipation, paresthesia, dizziness, dysgeusia	Nausea, constipation, headache, vomiting, dizziness, insomnia, dry mouth, diarrhea	Nausea, vomiting, constipation, diarrhea, headache, fatigue, dizziness, abdominal pain, increased lipase levels	Nausea, vomiting, constipation, diarrhea, headache, fatigue, dizziness, abdominal pain, increased lipase levels
Precautions	Pregnancy, use of MAOIs, hypersensitivity to sympathomimetic amines	Recommend a multivitamin when taking medication as it decreases intestinal absorption of fat‐soluble vitamins, can cause severe liver injury, avoid using with cyclosporine	May increase risk of pancreatitis, do not use with other GLP‐1 receptor agonists	Pregnancy, use of MAOIs, hypersensitivity to sympathomimetic amines	Drug or alcohol withdrawal, use of MAOIs, long‐term opioid use, pregnancy	May increase risk of pancreatitis, do not use with other GLP‐1 receptor agonists	May increase risk of pancreatitis, Do not use with other GLP‐1 receptor agonists
Contraindications	In patients with hypertension, myocardial infarction, arrhythmia, chronic kidney disease, mood disorders, glaucoma, hyperthyroidism.	In patients during pregnancy, with chronic malabsorption syndrome, cholestasis.	In patients with MEN2a and MEN2b, history of pancreatitis, T1DM, gastroparesis, family history of medullary thyroid cancer, Cr Cl < 30, eGFR < 30	In patients with hypertension, myocardial infarction, arrhythmia, chronic kidney disease, mood disorders, glaucoma, hyperthyroidism.	In patients with uncontrolled hypertension, seizure disorders, anorexia nervosa or bulimia.	In patients with MEN2a and MEN2b, history of pancreatitis, T1DM, gastroparesis, family history of medullary thyroid cancer, Cr Cl < 30, eGFR < 30	In patients with MEN2a and MEN2b, history of pancreatitis, T1DM, gastroparesis, family history of medullary thyroid cancer, Cr Cl < 30, eGFR < 30

Abbreviations: CNS, central nervous system; Cr Cl, creatinine clearance; GABA, gamma‐aminobutyric acid; eGFR, estimated glomerular filtration rate; GIP, glucose‐dependent insulinotropic peptides; GLP‐1 glucagon‐like peptide‐1; MAOI, monoamine oxidase inhibitor; T1DM, type 1 diabetes mellitus.

Side effects of orlistat include abdominal discomfort, fecal incontinence, steatorrhea, fecal urgency, and decreased absorption of fat‐soluble vitamins A, D, E, and K and minerals [23, 28]. Orlistat also reduces the absorption of medications such as cyclosporine, levothyroxine, warfarin, and anticonvulsants [23]. As orlistat can cause diarrhea, it may be beneficial for patients with constipation, including older adults, where constipation is commonly seen (26% for women and 16% for men) [26, 29]. In contrast, fecal incontinence is observed in up to 8.3% of older individuals and should be part of the evaluation when considering orlistat [23, 28, 30].

Because orlistat lacks sympathomimetic effects, its use is preferred in patients with cardiovascular diseases, such as coronary artery disease, and patients whose prevalence of cardiovascular disease rises with age [11]. Multivitamin supplementation is necessary as it increases intestinal motility, decreases absorption, inactivates lipases, and impairs the absorption of free fatty acids and fat‐soluble vitamins [11, 23]. We recommend that this medication be avoided in patients with food absorption disorders and in those who have undergone organ transplantation [11]. Orlistat is available in two formulations: 60 mg and 120 mg capsules. Orlistat can be started at any dose and should be taken immediately before, with, or within the first hour after a fat‐containing meal. The orlistat dose should be omitted if the older adult misses a meal or if it is a non‐fat‐containing meal. Always supplement with fat‐soluble vitamins A, D, E, and K when using orlistat [31] (Table 3).

### 3.5 Incretin Medications

Incretins, including glucagon‐like peptide‐1 receptor agonists (GLP1‐RAs) and dual GLP1‐RA/glucose‐dependent insulinotropic peptides (GIPs), are intestinal peptides secreted in response to nutrient intake, stimulating insulin secretion and slowing gastric emptying. They have been shown to improve satiety and reduce food intake and hunger perception, with mechanisms leading to weight loss. This class of medication was initially developed for glycemic control, yet the emerging generations have demonstrated significant weight loss, even in older adults, ranging from 15% to 25%. Currently, this class of medications is indicated for older adults with BMI ≥ 27 kg/m^2^ with at least one obesity‐related complication or with BMI ≥ 30 kg/m^2^ [11]. Incretin mimetics should be started at low doses in older adults to create tolerability and slowly titrate upward to the maximum dose tolerated. Here we discuss the different generations of incretins and the data relevant to older adults. AEs and guidance in prescribing these medications are provided in a section following their description.

#### 3.5.1 Liraglutide (Saxenda)

This medication was initially approved in 2014; yet, to our knowledge, few studies have been conducted in older adults with obesity. The Liraglutide: Effect and Action in Diabetes Evaluation of Cardiovascular Outcome Results study (LEADER) was a RCT that included 6183 participants aged 60–74 years and 836 participants over age 75 [32]. The outcomes included the effect of liraglutide on cardiovascular outcomes, major adverse cardiovascular events (MACE), myocardial infarction, cardiovascular death, and stroke. MACE were more frequently observed in the age group over 75 years when compared to the younger group of 60–74. In individuals aged 75 years and older, liraglutide reduced the risk of MACE by 34%. The risk reduction with liraglutide in all‐cause mortality when compared to placebo was 6% in the 60–74‐year group and 35% in the group over age 75 years. The use of liraglutide in older adults has been shown to reduce the risk of heart disease (14.3% of adults aged 65–74, and 24.2% of adults aged 75 and over) [32, 33].

The Satiety and Clinical Adiposity—Liraglutide Evidence (SCALE) Obesity and Prediabetes Trial, a 56‐week double‐blind trial, involved 3731 patients whose mean age was 45.1 years, did not have T2DM, and had BMI of 27 kg/m^2^ with untreated hypertension and/or dyslipidemia and/or had BMI of 30 kg/m^2^. Participants were randomly assigned to liraglutide 3.0 mg or placebo, and both groups received lifestyle modification counseling. The liraglutide group had a mean weight loss of 8.4 kg, and those in the placebo group had a mean weight loss of 2.8 kg (*p* < 0.001) [34]. The SCALE Diabetes Trial, a 56‐week double‐blind trial, consisted of participants with a mean age of 46 years, with T2DM, who were randomized to liraglutide 3.0 mg, liraglutide 1.8 mg, or placebo. Participants in the liraglutide 3 mg arm had a mean weight loss of 6.0%, liraglutide 1.8 mg resulted in a mean weight loss of 4.7%, and placebo had a mean weight loss of 4.7% [35]. Weight loss efficacy was reduced in this trial, although it was significant when compared to placebo. Data in both SCALE trials were limited for older adults; 7% of participants were aged 65 years and older, and only 0.5% were aged over 75 years. However, pooled analysis of participants showed similar mean weight loss in patients < 65 years old and > 65 years old. The SCALE Obesity and Prediabetes Trial randomized participants to liraglutide 3 mg or placebo. A mean body weight loss of 8% was observed with liraglutide after 56 weeks of treatment [23]. Liraglutide can be started at 0.6 mg SC daily, and the dose can be titrated weekly to 1.2 mg SC daily the second week, 1.8 mg SC daily the third week, 2.4 mg SC daily the fourth week, and up to 3.0 mg SC daily the fifth week. However, the use of liraglutide involves a subcutaneous daily dose, which can increase polypharmacy, which has a prevalence of 44.1% in older adults [36].

#### 3.5.2 Semaglutide (Wegovy)

This medication was approved in 2021, and its efficacy was proven through a series of clinical trials, termed the Semaglutide Treatment Effect in People with Obesity Program (STEP 1–5), that included more than 850 older adult individuals with overweight or obesity [19]. Briefly, STEP 1 randomized 1961 participants, without T2DM, to semaglutide 2.4 mg or placebo; mean weight loss was 14.9% vs. 2.4%, respectively [37]. Participants in the semaglutide group reported improved physical functioning and improvement in cardiometabolic risk factors, such as reduction in BMI, waist circumference, and diastolic and systolic blood pressure. Most participants in the semaglutide group lost ≥ 5%, 1047 (86.4%) of the participants; ≥ 10%, 838 (69.1%) of the participants; or > 15%, 612 (50.5%) of the participants of their baseline weight vs. 28 (4.9%) at week 68 (*p* < 0.001 for all). In the semaglutide group, the mean change from baseline body weight was 15.3 kg as compared to 2.6 kg in the placebo group [37]. STEP 2 randomized 1210 participants who had BMI of 30 kg/m^2^ or BMI of 27 kg/m^2^ with one or more obesity‐related comorbidities and T2DM (hemoglobin A1C [HbA1C] 7%–10%) to semaglutide 2.4 mg, semaglutide 1 mg, or placebo. At week 68, mean body weight from baseline with semaglutide 2.4 mg was 9.6% vs. 3.4% in the placebo group [38].

STEP 3 randomized participants to semaglutide 2.4 mg in addition to intensive behavioral therapy or placebo, and the mean weight reduction was 16% vs. 5.7%, respectively [39]. In the STEP 4 trial, 902 participants who had BMI of 30 kg/m^2^ or BMI of 27 kg/m^2^ with one or more obesity‐related comorbidities, without T2DM, were randomized to semaglutide 2.4 mg for 20 weeks, and mean weight loss was 10.6%. Further randomization of 803 of the participants who reached the semaglutide maintenance dose of 2.4 mg per week to 48 weeks continued semaglutide 2.4 mg or placebo. This trial showed a 7.9% mean weight reduction from the 20‐week weight when compared to placebo, in which participants regained 6.9% [40]. In the STEP 5 trial, 304 participants were randomized to semaglutide 2.4 mg weekly or placebo, and both groups received behavioral interventions. At week 104, the mean body weight from baseline in the group that received semaglutide was −15.2% vs. −2.6% in the placebo group. Fifty‐two participants in the semaglutide group achieved more than 20% mean body weight loss at week 104 vs. three participants from the placebo group. The semaglutide group also showed improved cardiometabolic outcomes in systolic blood pressure, diastolic blood pressure, HDL, LDL, total cholesterol, and triglycerides [41].

The Semaglutide Unabated Sustainability in Treatment of Type 2 Diabetes (SUSTAIN Trials 1–5) phase 3 trials pooled analysis demonstrated the efficacy and safety of semaglutide 0.5 mg and 1 mg vs. comparators, which included placebo, sitagliptin, exenatide, or insulin in non‐older adults < 65 years and older adults > 65 years with T2DM. The mean age of older adults participating was 69–70 years. Mean HbA1C decreased from baseline by 1.2%–1.5% and 1.5%–1.9% vs. 0%–0.9% in the non‐older adults (*n* = 3045) and in the older adults (*n* = 854) by 1.3%–1.5% and 1.2%–1.8% vs. 0.2%–1.0% with semaglutide 0.5 mg and 1 mg vs. comparators [42]. Across the five trials in older adults vs. non‐older adults, the reduced body weight with semaglutide 0.5 mg was 3.6‐4.6 kg vs. 3.3‐4.3 kg and with semaglutide 1 mg was 4.1‐6.7 kg vs. 4.6‐6.4 kg. Semaglutide reduced body weight comparably between older adults and non‐older adults. This pooled analysis also demonstrated that older adults lost more than 5% of mean body weight with semaglutide 0.5 mg (37%–59%) and 1 mg (40%–79%) vs. comparators (4%–17%) [42]. These findings also demonstrated cardiovascular benefit by improving cardiac function and attenuating atherothrombosis [23, 42]. Pooled data of these five trials also showed that most of the AEs were reported in severity as mild to moderate, and older adults reported more severe and profound AEs when using semaglutide. This also led to the premature discontinuation of semaglutide by older adults due to a higher incidence of GIAEs in this age group [42].

A post hoc analysis combining data from the SUSTAIN 6 and Peptide Innovation for Early Diabetes Treatment (PIONEER‐6) trials in older adults, mean age 65, with obesity and T2DM demonstrated that semaglutide led to risk reduction of experiencing the first MACE in patients with T2DM by 24% compared to placebo [43, 44].

The Semaglutide Effects on Cardiovascular Events in People with Overweight or Obesity (SELECT) trial randomized participants with a mean age of 61.6 years with preexisting cardiovascular disease, with BMI of 30 kg/m^2^ or BMI of 27 kg/m^2^ with one or more obesity‐related comorbidities and without T2DM to semaglutide 2.4 mg (*n* = 8803) or placebo (*n* = 8801) with follow‐up of 40 months. Overall, semaglutide showed a 20% risk reduction in primary cardiovascular outcomes, which included a composite of death from cardiovascular causes, nonfatal stroke, and nonfatal myocardial infarction in a time‐to‐first‐event analysis when compared to placebo [45]. A Spanish retrospective study that included 60 older adults with T2DM whose mean age was 76.5 years demonstrated that the use of once‐weekly semaglutide for 12 months induced a weight loss of 8.2 kg (SD ±5.3 kg) (*p* < 0.0001) and weight loss of ≥ 5% was achieved in 73%. Also, 67% of the participants achieved HbA1C < 7% [46].

The Evaluate Renal Function with Semaglutide Once Weekly trial (FLOW) randomized patients with chronic kidney disease (CKD), T2DM (HbA1C 10%), and a mean age of 66.6 years to semaglutide 1 mg or placebo and a mean follow‐up of 3.4 years. The primary outcome was to determine major kidney disease events, which included the onset of kidney failure (including dialysis, transplantation, eGFR 15 mL per minute per 1.73 m^2^), at least a 50% reduction in eGFR, or death from kidney‐related or cardiovascular causes. The risk of a major kidney disease event was reduced by 24% in the semaglutide group vs. placebo [47].

The Semaglutide Treatment Effect in People with Obesity and Heart Failure with Preserved Ejection Fraction and Diabetes Mellitus trial (STEP‐HFpEF DM) was a 52‐week randomized controlled study that included participants with a median age of 69 years, BMI ≥ 30 kg/m^2^, heart failure with preserved ejection fraction, and T2DM. This trial randomized participants in a 1:1 ratio to receive once‐weekly semaglutide 2.4 mg (*n* = 310) or placebo (*n* = 306). Primary endpoints of the study were percentage body weight change at week 52: −9.8 kg with semaglutide compared to −3.4 kg in the placebo group, and a change in the Kansas City Cardiomyopathy Questionnaire Clinical Summary Score (KCCQ‐CSS). The mean score at the beginning of the study was 59.35. In this questionnaire, scores range from 0 to 100, with higher scores indicating better quality of life. In the semaglutide group, an increase of +13.7 points was observed, compared to +6.4 points in the placebo group. Overall results indicated that the semaglutide group experienced significant weight loss, a reduction in physical limitations and heart failure‐related symptoms, and an improvement in exercise function compared to the placebo [48].

Semaglutide in older adults can be started at a dose of 0.25 mg SC weekly from week 1 to 4, 0.5 mg weekly from week 5 to 8, 1.0 mg SC weekly from week 9 to 12, 1.7 mg SC weekly from week 13 to 16, and up to 2.4 mg SC weekly from week 17 and thereafter.

#### 3.5.3 Tirzepatide (Zepbound)

This dual GLP1‐RA/GIP agonist was first approved in 2023 for weight loss. Indications for treatment are in patients with BMI ≥ 30 kg/m^2^ and in those with BMI ≥ 27 kg/m^2^ with one or more comorbidities. Trial results show participants losing up to 20% or more of mean body weight at 12 months [49]. SURMOUNT‐1 was a phase 3, double‐blind, randomized study that included 2539 participants, mean age 44.9 years, with BMI of 30 kg/m^2^ or higher, and those with BMI of 27 kg/m^2^ or greater with one or more comorbidities, excluding diabetes. Participants were randomized in a 1:1:1:1 ratio to receive once‐weekly subcutaneous tirzepatide (5 mg, 10 mg, or 15 mg) or placebo for 72 weeks, which included a 20‐week dose‐escalation period. At week 72, the mean percentage change in weight was −15.0%, −19.5%, −20.9%, and −3.1%, respectively. Weight reduction of 5% or more with tirzepatide vs. placebo was observed in 85% vs. 35%, 89% vs. 50%, and 91% vs. 57% of the participants with 5 mg, 10 mg, and 15 mg; the latter two doses saw 50% and 57% of participants have a reduction in body weight of ≥ 20% vs. 3% in the placebo arm. In this trial, tirzepatide improved cardiometabolic measures and provided significant and sustained weight loss [50]. SURMOUNT‐2, a phase 3, double‐blind, randomized study, included 938 participants with T2DM with mean HbA1C of 8.02%, obesity with a mean BMI of 36.1 kg/m^2^, and a mean age of 54 years. Participants were randomly assigned to receive tirzepatide 10 mg, tirzepatide 15 mg, or a placebo. At week 72, least‐squares mean change in body weight with tirzepatide 10 mg was −12.8%, tirzepatide 15 mg was −14.7%, and −3.2% with placebo [51].

SURMOUNT‐4 included participants with BMI > 30 kg/m^2^ without diabetes and with at least one weight‐related complication (i.e., hypertension, cardiovascular disease, obstructive sleep apnea [OSA], hyperlipidemia). There were 670 participants randomized to receive placebo vs. the maximum tolerated dose of tirzepatide (15 mg [92.7%] or 10 mg [7.3%]). The completion rate was 85.8%. For weeks 0–36 of the open‐label lead‐in period, 67 older adults (10%) were randomized. During this period, all participants (including older adults) achieved a 20.9% mean weight reduction. For weeks 36–88, 39 older adults (11.6%) were randomized to receive tirzepatide vs. 33 (9.9%) older adults who received placebo. All participants (including older adults) exhibited a mean weight change of −5.5% with tirzepatide vs. +14% with placebo. At week 88, participants receiving tirzepatide maintained at least 80% of the body weight lost during the first 36 weeks of the tirzepatide open‐label lead‐in period [52]. SURMOUNT‐4 outcomes demonstrated improved cardiometabolic parameters such as systolic and diastolic blood pressure, lipid levels, and glycemia, as well as reduction in waist circumference and BMI [52].

A pooled analysis of the SURPASS 1‐5 trials studied tirzepatide in adults, with BMI ≥ 30 kg/m^2^ and in those with BMI ≥ 27 kg/m^2^ with one or more comorbidities and with T2DM. Participants were randomized to weekly tirzepatide 5 mg, 10 mg, or 15 mg and comparator or placebo, and analysis was done for two subgroups: adults < 60 years old and > 60 years old [53].

Tirzepatide demonstrated a significant effect in reducing HbA1C and body weight. With tirzepatide 5 mg, the reduction of body weight was from −7 kg to −7.6 kg (< 60 years old) and −5.4 kg to −7.1 kg (> 60 years old); with tirzepatide 10 mg, it was −7.8 kg to −10.7 kg (< 60 years old) and −7.5 kg to −9.5 kg (> 60 years old); and with tirzepatide 15 mg, it was −9.5 kg to −12.9 kg (< 60 years old) and −8.8 kg to −11.7 kg (> 60 years old) compared to −0.7 kg to +2.3 kg in comparators. Tirzepatide was safe and efficacious in all the subgroups in these trials [53].

Tirzepatide's safety profile was also similar in adults < 65 years and > 65 years of age, as demonstrated in the East Asian SURPASS trial, which studied older adults with overweight or obesity and T2DM. Due to AEs, discontinuation of the medication was higher in the > 65 years group [54].

The SUMMIT RCT included 731 participants with BMI of at least 30 kg/m^2^ and heart failure with preserved ejection fraction (HFpEF) with or without T2DM. Participants (*n* = 731) were randomized to tirzepatide up to 15 mg weekly (starting dose 2.5 mg and increasing dose by 2.5 mg every week as tolerated to a maximum dose of 15 mg) vs. placebo (367 patients) for 52 weeks. The mean participant age was 65.2 years. The trial's two primary endpoints were a hierarchical composite of death from any cause or worsening heart failure events combined with changes in the KCCQ‐CSS and the 6‐min walk distance at week 52. The second endpoint was the change at week 52 in the 6‐min walk distance. Results at week 52 showed that 9.9% of the participants in the tirzepatide group had a worsening heart failure event or died from cardiovascular causes vs. 15.3% in the placebo group (*p* = 0.026). The KCCQ‐CSS mean increase was 19.5 points in the tirzepatide group vs. 12.7 points in the placebo group. At week 52, the mean body weight change was −13.9% in the tirzepatide group vs. 2.2% in the placebo group. The mean increase in the 6‐min walk in the tirzepatide group was 26.0 m vs. 10.1 m in the placebo group. Overall, the results over a median of 2 years of follow‐up demonstrated a reduced risk of worsening heart failure symptoms and improved health status in patients with heart failure with preserved ejection fraction, functional impairment, and obesity [55].

Tirzepatide has also been approved for the treatment of moderate to severe OSA as demonstrated in the SURMOUNT‐OSA trial. This was a 52‐week, phase 3, double‐blind trial, where participants' mean age was 47.9 years, with obesity and moderate to severe sleep apnea, and they were randomized to tirzepatide 10 mg, tirzepatide 15 mg, and placebo. Trial 1 enrolled participants without use of positive airway pressure (PAP) and trial 2 had use of PAP; the primary endpoint was the change of apnea‐hypopnea index (AHI). In trial 1, the mean change in AHI was −25.3 events per hour in the tirzepatide group vs. −5.3 events per hour with placebo. In trial 2, the mean change in AHI is −29.3 events per hour with tirzepatide and −5.5 events per hour with placebo [56].

Participants who received tirzepatide reduced the AHI and showed improved sleep‐related patient‐reported outcomes. Overall, the two trials demonstrate that tirzepatide was approximately five times more effective than the placebo [56].

Tirzepatide can be started in the older adult at a dose of 2.5 mg SC weekly from week 1 to 4; if well tolerated, you can increase the dose to 5.0 mg SC weekly from week 5 to 8. From week 9 to 12, you can increase the dose of tirzepatide to 7.5 mg SC weekly; then you can increase to 10.0 mg from week 13 to 16, and in week 17 to 20 you can increase to 12.5 mg SC weekly. The dose can go up to 15 mg SC weekly at week 21 and thereafter.

#### 3.5.4 Other Benefits of Incretins

The mechanism of action of incretins in slowing CKD progression is its multifaceted functions of GLP‐1s, such as anti‐inflammatory, antihyperglycemic, antihypertensive, antilipid, and endothelial function protective properties, leading to reduced structural and functional damage in renal tissues, positively affecting the glomeruli and tubules [57, 58]. Slowing cardiovascular risk can be achieved by incretins; as they have been shown to lower systolic blood pressure. They also inhibit macrophage activation in the endothelium, preventing atherosclerotic plaque formation [59].

Loss of weight leads to improvements in systolic and diastolic blood pressure, creating an opportunity to deprescribe antihypertensive medications. The association between cardiovascular complications and the use of AOMs was also recently described in a retrospective cohort study that included 34 billion Kythera Medicare claims from January 2020 to August 2022. This study had two cohorts: participants with obesity and not treated with AOMs (79,118 participants) and participants with obesity who were treated with AOMs (5926 participants). The mean age of participants in the AOMs cohort was 71.53 years, and for the non‐AOMs cohort, it was 73.67 years. The AOMs included were semaglutide and tirzepatide. A reduction in the incidence of atrial fibrillation by 35%, heart failure by 25%, arrhythmia by 18.6%, and peripheral vascular disease by 17% was seen in the participants in the AOMs cohort. An 8% risk reduction of cardiovascular disease was seen in the AOMs cohort [49].

Tirzepatide reduces triglycerides, LDL, and very low‐density lipoprotein cholesterol levels and significantly raises HDL levels as demonstrated in the SURPASS trials [53]. Emphasis on personalized treatment plans that consider cardiorenal risk and preferred weight loss treatment should be encouraged. Evidence has shown how semaglutide can reduce cardiovascular risk by 20% [45]. In an ongoing trial, SURPASS‐CVOT, the MACE of tirzepatide versus dulaglutide in participants with T2DM and atherosclerotic cardiovascular disease and tirzepatide‘s cardiovascular safety and efficacy are being measured [60]. Overall, incretins improve outcomes in cardiorenal disorders by improving outcomes in OSA, HFpEF, MACE, cardiovascular risk factors, myocardial infarction, cerebrovascular accident (CVA), cardiovascular death, T2DM, and metabolic dysfunction‐associated steatohepatitis (MASH).

Older adults with polypharmacy can benefit from incretin mimetics. The use of existing incretins can help prevent polypharmacy in older adults, as most of these medications are used once weekly. As older adults lose weight, improvements in other comorbidities can be observed, and deprescribing can also occur. A weight loss of greater than 5% can improve mobility, while a weight loss of greater than 10% can reduce the onset of T2DM, sleep apnea, MASH, cardiovascular disease, and overall mortality. Studies have shown that as patients show improvement in their metrics for T2DM, there are reductions or eliminations of insulin and/or other antidiabetic medications. Special consideration should be taken when GLP1‐RAs and GLP1‐RA/GIPs are prescribed to older adults who are also taking medications to treat T2DM, such as insulin and sulfonylureas, the latter of which is not recommended in older adults based on the Beers Criteria [61]. The use of GLP1‐RAs and GLP1‐RA/GIPs with hypoglycemic agents can cause episodes of hypoglycemia in older adults. However, there is a low risk of hypoglycemia in patients treated with incretins as monotherapy.

Incretins improve MASH as shown in the Effect of Semaglutide in Subjects with Non‐Cirrhotic Non‐Alcoholic Steatohepatitis (ESSENCE‐2) and SYNERGY NASH studies. ESSENCE‐2, a double‐blind, placebo‐controlled, phase 2 study, was a 72‐week trial that randomized 320 patients, with mean age 52.4 ± 10.8 years, to semaglutide 2.4 mg weekly vs. placebo. All participants had biopsy‐confirmed MASH and F1, F2, or F3 fibrosis. Patients in the semaglutide group showed superior resolution of and improvement in steatohepatitis without worsening fibrosis [62]. SYNERGY NASH is a double‐blind, placebo‐controlled, 52‐week, phase 2 study, with elegible participants 18–80 years old, mean age of participants 54.4 ± 11.3 years, with mean BMI of 36.1 ± 6.1 kg/m^2^. In this study, 190 patients with biopsy‐confirmed MASH and stage F2 and F3 fibrosis were randomized to tirzepatide 5 mg, 10 mg, or 15 mg vs. placebo. This trial also demonstrated resolution or improvement in steatohepatitis, with no effect on fibrosis [63].

Other benefits of incretins were demonstrated in a retrospective cohort study, *n* = 1,651,452, mean age 59.8 ± 15.1 years, whose participants had no prior diagnosis of obesity‐associated cancers (OACs), had T2DM, and were prescribed GLP‐1 s, insulin, or metformin. In patients who received GLP‐1 s, when compared to insulin, there was a significant reduction in the risk of having 10 out of 13 OACs: meningioma, multiple myeloma, hepatocellular carcinoma, gallbladder, pancreatic, ovarian, colorectal, esophageal, endometrial, and kidney cancers. Recent evidence has shown that GLP1‐RAs, compared with other antidiabetic medications, are associated with a statistically significant decreased risk for pancreatic cancer, with incidence hazard ratios ranging from 0.42 to 0.82; the reduction was greater in patients with obesity [11, 64, 65].

The SUSTAIN 1–5 pool analysis demonstrated the efficacy and safety of semaglutide in more than 850 older adults. Semaglutide 0.5 mg and 1 mg consistently improved body weight vs. comparators in older adults and also in younger patients, < 65 years, with T2DM [42]. SUSTAIN 6 demonstrated improved cardiovascular outcomes with semaglutide in patients whose average age was 65 years [43]. This could translate to GLP‐1 s being an effective and safe treatment for obesity and T2DM in older adults [42].

#### 3.5.5 Adverse Events

Incretins should be used carefully in older adults, given age‐related psychological and physiological factors. Adverse effects (AEs) of incretins include dyspepsia, nausea, vomiting, and diarrhea which can compromise the intake of essential nutrients in this population and can cause volume depletion. It is essential to recommend hydration to older adults undergoing treatment with incretins as dehydration in older adults can predispose them to acute kidney injury. Rarely pancreatic and thyroid cancer can occur.

The pooled analysis of the SUSTAIN trials 1–5 also looked to the GI events that lead to premature treatment discontinuation in older adults that received semaglutide 0.5 mg and 1 mg vs. comparator. The most common AEs were nausea, diarrhea, and vomiting and these were higher with semaglutide groups vs. comparators. Overall, GI side effects due to semaglutide 0.5 mg and 1 mg vs. comparators led to premature treatment discontinuation, and the incidence of AEs was higher in older adults‘ subgroup vs. the younger subgroup [42].

Special consideration should be given when using GLP1‐RAs due to decreased appetite and reduced intestinal motility, which impair digestion. Older adults may also face challenges with chewing and swallowing, which can compromise digestion. Additionally, diarrhea and/or constipation may pose significant challenges, as older adults tend to drink less water due to decreased thirst receptors. Reduced urinary concentrating ability of the kidney, often seen in older adults, may be affected as well. These conditions can be further exacerbated with cognitive impairment, delirium, and depression [66]. Furthermore, limited water intake can heighten the risk of constipation and impaction.

#### 3.5.6 Special Considerations

GLP1‐RAs and GLP‐1RA/GIPs are medications that are contraindicated in older adults with a past medical history of multiple endocrine neoplasia syndrome type 2 (MEN‐2) and a family history of medullary thyroid cancer (MTC) or in patients with a history of pancreatitis. GLP1‐RAs and GIPs are also contraindicated in older adults with type 1 diabetes (DMT1), diabetic ketoacidosis, and gastroparesis. While less common among older adults, we generally avoid GLP1‐RAs and GLP1‐RA/GIPs with inadequate food intake, malnutrition, or eating disorders such as anorexia and or bulimia. Finally, skin reactions can occur at injection sites, so it is essential to remind the patient or caregiver to rotate injection sites to prevent this occurrence.

In older adults with cognitive impairment, AOMs should be cautiously prescribed, as adherence could be problematic. We recommend that the AOMs preferred in older adults with mild, moderate, and severe cognitive impairment are the GLP1‐RAs and GLP1‐RA/GIPs (most given once weekly). Emerging evidence suggests that incretins may reduce the rate of cognitive decline. The Evaluating Liraglutide in Alzheimer‘s Disease (ELAD) clinical trial compared liraglutide to DPP‐4 inhibitors (DPP‐4i) in reducing the risk of Alzheimer‘s disease and related dementias (ADRD). The study included participants aged 70 and older who did not have ADRD and were followed for a mean of 3 years. The incidence of ADRD was 196 participants in the liraglutide group and 3125 participants in the DPP‐4i group. The study showed that liraglutide is associated with a decreased ADRD risk when compared to DPP‐4i [67].

## 4 Functional Status and Musculoskeletal Effects

Aging is associated with increased adipose tissue and visceral adipose tissue, which promote proinflammatory factors that impair insulin sensitivity, ultimately leading to decreased muscle growth and further muscle wasting as well as sarcopenia. Aging is also associated with an increased infiltration of skeletal muscle by adipose tissue, or myosteatosis, which impairs muscle function and reduces functional capacity and mobility. Sarcopenia is characterized by decreased muscle mass, strength, and function and it is common in older adults with obesity due to the metabolic changes seen with aging, sedentary lifestyle, and adipose tissue derangements. Sarcopenic obesity is referred to as an excess in adipose tissue with low muscle mass and function. However, not all older adults with obesity develop sarcopenia. Sarcopenic obesity has a prevalence that ranges broadly but approximates 28.3% in adults > 60 years of age [68].

Age‐related changes in older adults include increased insulin resistance, decreased energy expenditure, decreased protein synthesis, increased adiposity with increased inflammation, increased lipotoxicity, ectopic fat deposition, decreased muscle strength, and reduced muscle mass. As patients lose weight, they lose weight from various parts of their bodies, including total body water, fat, bone, and muscle mass, with a risk of experiencing more muscle loss than fat mass loss, which can lead to sarcopenia.

In the Look AHEAD study, participants with overweight, obesity, and T2DM, with BMI > 25 kg/m^2^ or > 27 kg/m^2^ if receiving insulin and an age range of 45–76, were randomized to lifestyle intervention (ILI) or diabetes support and education. Dual energy X‐ray absorptiometry (DXA) was used to measure changes in body composition, specifically in fat mass and lean mass. At 8 years of intervention, weight loss was greater in the intervention group (4.0 kg ±0.4 kg vs. 2.3 kg ±0.4 kg). Comparison group weight loss was mainly lean mass (2.3 kg ±0.17 kg). There was a significant difference in weight loss between the two groups. In both groups, a gain in fat mass was observed, accompanied by a loss of muscle mass [69].

Weight loss might accelerate age‐related loss of muscle and bone mass and result in sarcopenia and osteopenia. A clinical trial by Villareal et al. included 160 older adults with obesity. Participants were randomized to a weight management program and assigned to either aerobic training, resistance training, or combined aerobic and resistance training or to a control group (no weight management or exercise program). At 6 months, the Physical Performance Test Score, which is a measure that ranges from 0 to 36 points, with higher scores meaning better performance, showed better results in the combination group when compared to the resistance and aerobic group. Strength increased more in the combination and resistance group than in the aerobic group. Body weight decreased by 9% in all exercise groups and did not change significantly in the control group. Lean mass decreased less in the combination group and resistance group than the aerobic group, 3% decrease and, 2% decrease, respectively, compared to a 5% decrease. Bone mineral density at the total hip decreased by 1% in the combination group and by 0.5% in the resistance group, compared to a 3% decrease in the aerobic group (*p* < 0.05 for all comparisons) [70]. A combination of aerobic resistance training and diet led to weight loss‐induced lean mass but improved strength in older adults with obesity. In a randomized controlled trial conducted in post‐menopausal women, for every 1 kg of fat lost during the weight loss period, there was a 0.26 kg loss of lean mass. For every 1 kg of fat regained over the following year there was only 0.12 kg lean mass regained [71].

Patients with sarcopenic obesity have poor functional muscle outcomes as demonstrated in 599 participants with a mean age of 51.3 ± 14.2 years treated in a multidisciplinary and wellness rural center that had a bioelectrical impedance analysis (BIA) at intake using sex‐specific cut points for appendicular lean mass (ALM < 19.75 kg in men and < 15.02 kg in women defined sarcopenia) and body fat (defined as elevated body fat exceeding 25% and 35% in men and women, respectively, and > 88 cm and > 102 cm in waist circumference). Grip strength was lower (25.1 ± 8.0 vs. 30.5 ± 11.3 kg; *p* < 0.001) and sit‐to‐stand times were longer (12.4 ± 4.4 vs. 10.8 ±4.6s; *p* = 0.03) in patients with sarcopenic obesity vs. those without it [72].

A prospective study in nine older adults with T2DM, obesity, and/or overweight receiving liraglutide 3 mg per day for 24 weeks showed a median decrease of weight (−2 kg), fat mass (−1.498 kg), and android fat (−0.9%) and an increase of skeletal muscle index (+0.03 kg/m^2^) [73]. Body composition analysis with DXA scan in 140 participants of the STEP 1 trial who were 18 years and older with overweight or obesity without T2DM, mean weight 98.4 kg, mean BMI 34.8 kg/m^2^, randomized to semaglutide 2.4 mg SC weekly for 68 weeks vs. placebo, showed percentage change in body weight from baseline of −15% with semaglutide vs. −3.6% with placebo. Reductions from baseline with semaglutide in total fat mass (−19.3%) and regional visceral fat mass (−27.4%) led to a 3.5% point and 2.0% point reductions in the proportions of total fat mass and visceral fat mass, respectively. The total lean body mass decreased from baseline (−9.7%); however, the proportion relative to total body mass increased by 3.0% points. Overall, an increase in lean body mass:fat mass ratio was seen with semaglutide with increasing weight loss from baseline to week 68 [74]. An absolute reduction of 10.4 kg total fat mass and 6.9 kg total body lean mass was seen corresponding to a proportional body weight reduction of 60% fat mass and 40% lean mass. A randomized study by Heise et al. investigated the effects of tirzepatide, semaglutide, and placebo on body composition, appetite, and energy intake in adults with mean age 62 and with T2DM over 28 weeks. Total body weight was higher in the tirzepatide group when compared to semaglutide and placebo (*p* < 0.001), and fat‐free mass weight loss was higher with tirzepatide (−1.5 kg [−2.3, −0.7]; *p* < 0.001) and semaglutide (−0.8 kg [−1.5, −0.1]; *p* = 0.018) [75].

As humans age, there is an increase in fat mass accompanied by a gradual decline in lean mass, particularly muscle mass. The SURMOUNT‐1 DXA substudy demonstrated average weight changes of −21.3% with tirzepatide compared with −5.3% with placebo. DXA scans were conducted in 160 participants with a mean BMI of 38.0 kg/m^2^ at the beginning of the study and at week 72. Fat mass and lean mass from baseline to week 72 with tirzepatide were −33.9% and −0.9% vs. placebo −8.2% and −2.6%, respectively (*p* < 0.001 for all comparisons). In this study, fat mass loss compared to lean mass loss was 75%‐25% in the tirzepatide group. In older adults, tirzepatide was not associated with a proportionally greater loss of lean tissue. However, it is important to note that the sample size was small (*n* = 160 out of the 2539 participants in SURMOUNT‐1 trial). In this study, participants aged 65 and older, despite an increase in the percentage of lean mass, experienced a decrease in absolute lean mass of 6 kg, which translates to approximately 10 years of skeletal muscle aging [76]. Thus, in older adults, preserving muscle is vital as sarcopenia can increase functional limitations, risk of falls, risk of mortality, risk of cognitive impairment, and decrease in quality of life [72].

While lifestyle‐based weight loss studies have demonstrated improved quality of life, weight loss from newer incretins may also impact physical function. While existing studies of AOMs have limited the inclusion of older adults to evaluate these outcomes, the impact of fat and muscle loss on the hypothesized improvements in ectopic adipose tissue deposits and reduced myosteatosis, which ultimately lead to improved function, remains unclear [68]. The STEP 1 trial showed that physical functioning improved significantly at week 68 in the semaglutide group, as shown by the Short Form Health Survey, 36‐item version 2.0 (SF‐36v2) physical functioning scores (norm‐based scores ranging from 19.03–57.60) vs. placebo (*p* < 0.001) [74]. Also, the Impact of Weight on Quality of Life‐Lite Clinical Trials Version (IWQOL‐Lite‐CT) physical function scores significantly improved in the semaglutide group vs. the placebo group (*p* < 0.001) Although improved functional outcomes were seen in the participants (mean age 46), participation of older adults and outcome results with semaglutide are limited in this age group. Overall, the STEP trials demonstrated improved outcomes in the SF‐36 in STEP 1 and 2 and improvement in the IWQOL‐Lite‐CT in STEP 1, 2, and 4 [77]. The SURMOUNT‐1 trial showed improved functional scores in the SF‐36 in the tirzepatide group when compared to placebo at week 72 [50].

Newer AOMs such as semaglutide and tirzepatide carry the potential risk for older adults to develop loss of muscle as an AE [19]. There is an importance in identifying sarcopenic obesity due to its association with an increased risk of metabolic dysfunction, geriatric syndromes, and death [68, 72]. Screening older adults for sarcopenia is indicated in those with high BMI or waist circumference, based on ethnic cut points. Surrogate parameters for sarcopenia, such as having the clinical suspicion or through questionnaires, e.g., strength, assistance in walking, rising from a chair, climbing stairs, and falls or sarcopenia questionnaires (SARC‐F), should be considered. Diagnosis can be evaluated by evaluating altered skeletal muscle functional parameters with the HGS or the Chair Stand Test, and if these parameters demonstrate an abnormality, the clinician should assess for body composition. Body composition can be evaluated by appendicular lean mass adjusted by weight (ALM/W) by DXA or as skeletal muscle mass adjusted by weight (SMM/W) by BIA. To determine the presence of sarcopenia, both altered skeletal muscle function and body composition must be present. After sarcopenia is confirmed, a two‐level staging should be performed. Stage 1 is when no complications are attributable to altered body composition and skeletal muscle parameters, and Stage 2 is the presence of at least one complication attributable to altered body composition and skeletal muscle functional parameters such as metabolic diseases, cardiovascular diseases, and disabilities by high fat mass and/or low muscle mass.

Older adults who are losing weight experience changes in fat, muscle, and bone that alter the physiological impact within metabolically active tissues, leading to improved metabolic consequences but also increasing the risk of developing frailty and osteoporosis [78]. A meta‐analysis of the effects of GLP‐1s on fracture risk showed that exenatide had the highest safety and lower risk of fracture (0.07%) when compared to dulaglutide (1.04%), liraglutide (1.39%), albiglutide (5.61%), lixisenatide (8.07%), and semaglutide (18.72%) [79]. Overall, this and other meta‐analyses demonstrated that GLP‐1s could reduce the risk of fractures in patients with T2DM [79]. Alternatively, other meta‐analyses demonstrated that GLP1‐RAs showed potential benefits for bone health in the treatment of T2DM. The study also concluded that the longer patients were on GLP1‐RAs, the more significant improvements were seen in lumbar bone mass density [80].

## 5 Multicomponent Interventions When Prescribing AOMs in Older Adults

In this section we provide a brief overview of concomitant lifestyle, nutrition, and exercise‐based interventions that can be co‐prescribed with AOMs for older adults. This is important when considering the newer incretin therapies that lead to weight loss. These interventions are thoroughly described and reviewed elsewhere [81, 82].

### 5.1 Nutritional Interventions

In older adults, hypocaloric diets can reduce adipose tissue and negatively affect muscle mass. Older adults pursuing weight loss treatment with AOMs should have a minimal protein intake of 1.0–1.2 g/kg body weight. Others recommend a higher protein intake of 1.2–1.5 g/kg body weight due to their reduced anabolic response to protein. A deficit of 200–700 kcal of reduced energy intake per day is recommended, targeting weight loss of 0.5–1 kg per week or 8%–10% of initial body weight at 6 months. In older adults, essential amino acids are essential for protein synthesis, and leucine supplementation with 2.0–2.5 g per day is associated with increased muscle protein synthesis. In older adults with extensive stage IV/V CKD and obesity, careful consideration of protein supplementation should be followed, and protein intake should be limited to 0.6–0.8 g/kg of body weight [83, 84].

Incretins lead to increased satiety, and thus, intake of the daily protein requirement in one meal can be uncomfortable, given the side effects of delayed gastric emptying, nausea, and vomiting. Smaller portions throughout the day can be considered to fulfill daily protein requirements and maintain an appropriate intake during weight loss efforts. Delayed gastric emptying can cause constipation, and in older adults, less food intake can cause less fiber consumption and increase the risk for constipation. A daily requirement of 25–30 g of fiber per day is encouraged either through diet or supplementation. Delayed gastric emptying can impair the absorption of vitamin B12; thus, supplementation and regular monitoring are encouraged.

As weight loss has been associated with decreased bone mineral density, calcium 1,000–1,200 mg per day is recommended in older adults, particularly those at increased risk of osteoporosis [85]. Also, older adults are known to have vitamin D insufficiency and deficiency. In older adults taking AOMs, supplementation with vitamin D is encouraged, as vitamin D also helps with calcium absorption. In clinical practice, older adults taking AOMs should consult with a registered dietitian to ensure they maintain appropriate eating habits while taking these medications. It is essential to note that older adults prescribed orlistat are at risk of deficiencies in fat‐soluble vitamins A, D, E, and K.

A 12‐week RCT in older adults with obesity demonstrated that weight loss with very low‐calorie diets (VLCDs) combined with exercise improved HbA1C, cholesterol, triglycerides, and systolic blood pressure, overall improving cardiovascular outcomes and diabetes [86]. However, sustainability with VLCDs in older adults is questionable due to an increased risk of micronutrient deficiency, decreased bone mineral density, disordered eating behavior, and overall muscle mass loss.

### 5.2 Exercise Interventions

Older adults with BMI > 40 kg/m^2^ who experience rapid weight loss with AOMs may see dramatic muscle loss that can lead to sarcopenic obesity. Resistance exercises can help increase muscle mass. Recommended for older and frail adults are slow and fast velocity exercises consisting of 1–2 sets of 8–12 repetitions each at 65% of one repetition maximum and progressing to 2–3 sets of 10–15 repetitions each at 75% of one repetition maximum. Aerobic exercises can improve muscle function and induce loss of both visceral and total adipose tissue. Achieving 65% of the peak heart rate and progressing to 70%–85% of peak heart rate over the duration of the exercise regimen is recommended. Both aerobic and resistance exercise can improve muscle mass and function [68].

## 6 Ongoing Monitoring Using AOMs

Long‐term success of any weight loss program in older adults depends on building rapport and educating the patient about the importance of healthy weight loss for preventing or improving chronic diseases, such as hypertension, T2DM, hyperlipidemia, and OSA. A weight loss of 5%–10% of total body weight can lead to health benefits; and while newer generations of incretins exceed this, the optimal balance between losing weight and improving function is unknown. A major ongoing challenge in clinical geriatrics is polypharmacy. The benefit of AOMs is that if treatment helps patients lose weight, it can help improve other health conditions and lead to deprescribing, thus reducing the number or decreasing medications for older adults. Currently, there are no prescribing guidelines tailored to older adults. While BMI is often used to assess for obesity, it has poor diagnostic accuracy, particularly in older adults.

Where able, using techniques to assess body composition can be helpful. Unfortunately, gold standard techniques such as CT or MRI are costly and impractical. The United States Prevention Services Task Force (USPSTF) recommends dual energy x‐ray absorptiometry DXA scans to measure bone density to prevent osteoporotic fractures for women at age 65 and men at age 80, or at a younger age if there is an increased risk of osteoporosis. DXA scans can also measure body composition, and if effectively implemented in clinical workflows, these scans could be a helpful monitoring tool during treatment for obesity among older adults. Bioimpedance analysis can also assess body composition, which measures the water proportion of body weight and bone mass. Newer technology, such as Visual Body Composition (VBC), can estimate body fat percentage accurately and without significant bias from DXA scans through smartphones. In this trial, VBC measurement of body fat was compared to professional bioimpedance systems and air displacement plethysmography, and VBC performance was superior to these other methods. In the clinical setting, it is highly encouraged to evaluate for sarcopenic obesity in older adults in treatment for obesity with AOMs, per the Sarcopenic Obesity Global Leadership Initiative [68]. Laboratory tests to consider when enrolling older adults in weight management and the use of AOMs include a complete blood count, complete metabolic panel, lipid panel, HbA1C, thyroid stimulating hormone, free T4, and vitamin D levels.

Overall, the optimal patient for AOMs is an older adult with BMI > 30 kg/m^2^ or > 27 kg/m^2^ and one or more obesity‐related comorbidities who has not been able to lose weight with lifestyle changes alone, including diet and exercise. Personal health care goals should be considered in older adults who start AOMs; an individualized benefit–risk assessment and shared decision making should be done. AOMs for the treatment of obesity in older adults can improve functionality, quality of life, and independence. Older adults with AOMs should be motivated and have no medical contraindications to be on these medications. Older adults can benefit from incretins to lose 15%–20% of their weight from baseline. As demonstrated in the trials described here, these medications can improve cardiovascular outcomes, hypertension, hyperlipidemia, metabolic associated liver disease (MALD)/metabolic associated steatohepatitis (MASH), OSA, and CKD [56, 57, 62, 63]. Further trials that emphasize frailty and muscle mass in older adults are needed.

## 7 Conclusion

Obesity rates have risen dramatically, including among older adults, but gaps in knowledge in the treatment of obesity in older adults persist. Most studies that have been conducted include participants aged 18–65; future research needs to include adults 65 years of age and older, particularly those who are 75 and older, who are frequently prescribed weight loss medications. Long‐term use efficiency studies of newer AOMs in older adults are urgently needed. Research on the impact of AOMs on quality of life and patient outcomes is also required. Defining treatment guidelines for obesity in older adults has been challenging to establish, focuses solely on data from behavioral trials, and has not been updated since 2005. Metabolic, physiological, and behavioral changes that occur with aging make it essential to tailor individual healthy lifestyle plans to each older adult. From a policy standpoint, the importance of coverage of AOMs for older adults represents an urgent opportunity for this population with a chronic disease. Incretins such as semaglutide and tirzepatide have considerable potential in this population to reduce complications of obesity and improve cardiovascular outcomes, and while they have demonstrated efforts in recruiting older adults, greater participation of older adults in clinical trials is needed.

## Conflicts of Interest

The authors declare no conflicts of interest.

## References

[1] G. A. Bray , K. K. Kim , J. P. H. Wilding , and World Obesity Federation , “Obesity: A Chronic Relapsing Progressive Disease Process. A Position Statement of the World Obesity Federation,” Obesity Reviews 18, no. 7 (2017): 715–723, 10.1111/obr.12551. 28489290

[2] J. Batsis , C. Haudenschild , A. Kahkoska , et al., The Chronic Disease of Obesity (Gerontological Society of America, 2023).

[3] M. Jura and L. P. Kozak , “Obesity and Related Consequences to Ageing,” Age (Dordr) 38, no. 1 (2016): 23, 10.1007/s11357-016-9884-3. 26846415 PMC5005878

[4] J. A. Batsis and A. B. Zagaria , “Addressing Obesity in Aging Patients,” Medical Clinics of North America 102, no. 1 (2018): 65–85, 10.1016/j.mcna.2017.08.007. 29156188 PMC5724972

[5] J. A. Harris and N. G. Castle , “Obesity and Nursing Home Care in the United States: A Systematic Review,” Gerontologist 59, no. 3 (2019): e196–e206. 29253135 10.1093/geront/gnx128 PMC6524472

[6] W. T. Garvey , J. I. Mechanick , E. M. Brett , et al., “Comprehensive Clinical Practice Guidelines for Medical Care of Patients With Obesity,” Endocrine Practice 22, no. S3 (2016): 1–203, 10.4158/EP161365.GL. 27219496

[7] “Report: Adults 50 and Older Need More Physical Activity,” Centers for Disease Control and Prevention, published April 23 2024, https://www.cdc.gov/physical‐activity/php/reports/adults‐50‐and‐older.html.

[8] L. Perreault , C. M. Apovian , and T. J. Reid , “Obesity in Adults: Overview of Management,” UpToDate, last modified September 17, 2025, https://www.uptodate.com/contents/obesity‐in‐adults‐overview‐of‐management.

[9] M. K. Keating , R. K. Woodruff , and E. M. Saner , “Management of Obesity: Office‐Based Strategies,” American Family Physician 110, no. 2 (2024): 145–156. 39172672

[10] E. Grunvald , R. Shah , R. Hernaez , et al., “AGA Clinical Practice Guideline on Pharmacological Interventions for Adults With Obesity,” Gastroenterology 163, no. 5 (2022): 1198–1225, 10.1053/j.gastro.2022.08.045. 36273831

[11] S. Abdi Beshir , A. Ahmed Elnour , A. Soorya , et al., “A Narrative Review of Approved and Emerging Anti‐Obesity Medications,” Saudi Pharmaceutical Journal 31, no. 10 (2023): 101757, 10.1016/j.jsps.2023.101757. 37712012 PMC10497995

[12] C. Apovian , L. Aronne , D. H. Bessesen , et al., “Pharmacological Management of Obesity: An Endocrine Society Clinical Practice Guideline,” Journal of Clinical Endocrinology & Metabolism 100 (2015): 342–362, 10.1210/jc.2014-3415. 25590212

[13] B. G. Tchang , M. Aras , R. B. Kumar , et al., “Pharmacologic Treatment of Overweight and Obesity in Adults,” In Endotext [Internet], ed. K. R. Feingold, S. F. Ahmed, B. Anawalt, et al. (MDText.com, Inc., 2024), https://www.ncbi.nlm.nih.gov/books/NBK279038/.

[14] S. B. Heymsfield and T. A. Wadden , “Mechanisms, Pathophysiology, and Management of Obesity,” New England Journal of Medicine 376 (2017): 254–266, 10.1056/NEJMra1514009. 28099824

[15] B. Matyjaszek‐Matuszek , A. Szafraniec , and D. Porada , “Pharmacotherapy of Obesity‐State of the Art,” Endokrynologia Polska 69, no. 4 (2018): 448–466, 10.5603/EP.2018.0048. 30209803

[16] J. F. Munro , A. C. MacCuish , E. M. Wilson , and L. J. Duncan , “Comparison of Continuous and Intermittent Anorectic Therapy in Obesity,” BMJ 1, no. 5588 (1968): 352–354, 10.1136/bmj.1.5588.352. 15508204 PMC1984840

[17] J. Jordan , A. Astrup , S. Engeli , K. Narkiewicz , W. W. Day , and N. Finer , “Cardiovascular Effects of Phentermine and Topiramate: A New Drug Combination for the Treatment of Obesity,” Journal of Hypertension 32, no. 6 (2014): 1178–1188, 10.1097/HJH.0000000000000145. 24621808 PMC4011567

[18] E. P. Terlizzi and M. A. Villarroel , “Symptoms of Generalized Anxiety Disorder Among Adults: United States, 2019,” NCHS Data Brief no. 378 (2020): 1–8. 33054928

[19] A. Gavras and J. A. Batsis , “Medical Weight Loss in Older Persons With Obesity,” Clinical Obesity 14, no. 5 (2024): e12684, 10.1111/cob.12684. 38924367 PMC11570349

[20] K. M. Gadde , D. B. Allison , D. H. Ryan , et al., “Effects of Low‐Dose, Controlled‐Release, Phentermine Plus Topiramate Combination on Weight and Associated Comorbidities in Overweight and Obese Adults (CONQUER): A Randomised, Placebo‐Controlled, Phase 3 Trial,” Lancet 377, no. 9774 (2011): 1341–1352, 10.1016/S0140-6736(11)60205-5. 21481449

[21] T. Garvey , D. Ryan , M. Look , et al., “Two‐Year Sustained Weight Loss and Metabolic Benefits With Controlled‐Release Phentermine/Topiramate in Obese and Overweight Adults (SEQUEL): A Randomized, Placebo‐Controlled, Phase 3 Extension Study,” American Journal of Clinical Nutrition 95, no. 2 (2012): 297–308. 22158731 10.3945/ajcn.111.024927 PMC3260065

[22] D. B. Allison , K. M. Gadde , W. T. Garvey , et al., “Controlled‐Release Phentermine/Topiramate in Severely Obese Adults: A Randomized Controlled Trial (EQUIP),” Obesity (Silver Spring) 20 (2012): 330–342, 10.1038/oby.2011.330. 22051941 PMC3270297

[23] H. Henney , J. P. H. Wilding , U. Alam , et al., “Obesity Pharmacotherapy in Older Adults: A Narrative Review of Evidence,” International Journal of Obesity (Lond) 49 (2025): 369–380, 10.1038/s41366-024-01529-z. PMC11971046 38710803

[24] F. L. Greenway , K. Fujioka , R. A. Plodkowski , et al., “Effect of Naltrexone Plus Bupropion on Weight Loss in Overweight and Obese Adults (COR‐I): A Multicentre, Randomised, Double‐Blind, Placebo‐Controlled, Phase 3 Trial,” Lancet 376, no. 9741 (2010): 595–605, 10.1016/S0140-6736(10)60888-4. 20673995

[25] M. M. Sherman , S. Ungureanu , and J. A. Rey , “Naltrexone/Bupropion ER (Contrave): Newly Approved Treatment Option for Chronic Weight Management in Obese Adults,” P & T 41, no. 3 (2016): 164–172. 26957883 PMC4771085

[26] S. Z. Yanovski and J. A. Yanovski , “Long‐Term Drug Treatment for Obesity: A Systematic and Clinical Review,” JAMA 311, no. 1 (2014): 74–86, 10.1001/jama.2013.281361. 24231879 PMC3928674

[27] J. S. Torgerson , J. Hauptman , M. N. Boldrin , and L. Sjöström , “XENical in the Prevention of Diabetes in Obese Subjects (XENDOS) Study: A Randomized Study of Orlistat as an Adjunct to Lifestyle Changes for the Prevention of Type 2 Diabetes in Obese Patients,” Diabetes Care 27 (2004): 155–161, 10.2337/diacare.27.1.155. 14693982

[28] D. T. Villareal , C. M. Apovian , R. F. Kushner , S. Klein , American Society for Nutrition , and NAASO, The Obesity Society , “Obesity in Older Adults: Technical Review and Position Statement of the American Society for Nutrition and NAASO, the Obesity Society,” Obesity Research 13, no. 11 (2005): 1849–1863, 10.1038/oby.2005.228. 16339115

[29] B. G. Schuster , L. Kosar , and R. Kamrul , “Constipation in Older Adults: Stepwise Approach to Keep Things Moving,” Canadian Family Physician 61, no. 2 (2015): 152–158. 25676646 PMC4325863

[30] W. E. Whitehead , L. Borrud , P. S. Goode , et al., “Fecal Incontinence in US Adults: Epidemiology and Risk Factors,” Gastroenterology 137, no. 2 (2009): 512–517, 10.1053/j.gastro.2009.04.054. 19410574 PMC2748224

[31] A. B. Bansal , P. Patel , and Y. Al Khalili , “Orlistat,” In StatPearls [Internet] (StatPearls Publishing, 2024), https://www.ncbi.nlm.nih.gov/books/NBK542202/. 31194359

[32] M. P. Gilbert , S. C. Bain , E. Franek , et al., “Effect of Liraglutide on Cardiovascular Outcomes in Elderly Patients: A Post Hoc Analysis of a Randomized Controlled Trial,” Annals of Internal Medicine 170, no. 6 (2019): 423–426, 10.7326/M18-1569. 30508430

[33] M. Heron , “Deaths: Leading Causes for 2019,” National Vital Statistics Reports 70, no. 9 (2021): 1–114. 34520342

[34] X. Pi‐Sunyer , A. Astrup , K. Fujioka , et al., “A Randomized, Controlled Trial of 3.0 Mg of Liraglutide in Weight Management,” New England Journal of Medicine 373, no. 1 (2015): 11–22, 10.1056/NEJMoa1411892. 26132939

[35] M. J. Davies , R. Bergenstal , B. Bode , et al., “Efficacy of Liraglutide for Weight Loss Among Patients With Type 2 Diabetes: The SCALE Diabetes Randomized Clinical Trial,” JAMA 314, no. 7 (2015): 687–699, 10.1001/jama.2015.9676. 26284720

[36] X. Wang , K. Liu , K. Shirai , et al., “Prevalence and Trends of Polypharmacy in U.S. Adults, 1999‐2018,” Global Health Research and Policy 8, no. 1 (2023): 25, 10.1186/s41256-023-00311-4. 37434230 PMC10337167

[37] A. Fornes , J. Huff , R. I. Pritchard , and M. Godfrey , “Once‐Weekly Semaglutide for Weight Management: A Clinical Review,” Journal of Pharmacy Technology 38, no. 4 (2022): 239–246, 10.1177/87551225221092681. PMC9272494 35832567

[38] M. Davies , L. Færch , O. K. Jeppesen , et al., “Semaglutide 2·4 Mg Once a Week in Adults With Overweight or Obesity, and Type 2 Diabetes (STEP 2): A Randomised, Double‐Blind, Double‐Dummy, Placebo‐Controlled, Phase 3 Trial,” Lancet 397, no. 10278 (2021): 971–984, 10.1016/S0140-6736(21)00213-0. 33667417

[39] T. A. Wadden , T. S. Bailey , L. K. Billings , et al., “Effect of Subcutaneous Semaglutide vs Placebo as an Adjunct to Intensive Behavioral Therapy on Body Weight in Adults With Overweight or Obesity: The STEP 3 Randomized Clinical Trial,” JAMA 325, no. 14 (2021): 1403–1413, 10.1001/jama.2021.1831. 33625476 PMC7905697

[40] D. Rubino , N. Abrahamsson , M. Davies , et al., “Effect of Continued Weekly Subcutaneous Semaglutide vs Placebo on Weight Loss Maintenance in Adults With Overweight or Obesity: The STEP 4 Randomized Clinical Trial,” JAMA 325, no. 14 (2021): 1414–1425, 10.1001/jama.2021.3224. 33755728 PMC7988425

[41] W. T. Garvey , R. L. Batterham , M. Bhatta , et al., “Two‐Year Effects of Semaglutide in Adults With Overweight or Obesity: The STEP 5 Trial,” Nature Medicine 28, no. 10 (2022): 2083–2091, 10.1038/s41591-022-02026-4. PMC9556320 36216945

[42] M. Warren , L. Chaykin , D. Trachtenbarg , G. Nayak , N. Wijayasinghe , and B. Cariou , “Semaglutide as a Therapeutic Option for Elderly Patients With Type 2 Diabetes: Pooled Analysis of the SUSTAIN 1‐5 Trials,” Diabetes, Obesity & Metabolism 20, no. 9 (2018): 2291–2297, 10.1111/dom.13331. PMC6099273 29687620

[43] S. Verma , J. P. David , L. A. Leiter , M. M. Michelsen , S. Rasmussen , and D. L. Bhatt , “Semaglutide Reduces the Risk of Major Adverse Cardiovascular Events Consistently Across Baseline Triglyceride Levels in Patients With Type 2 Diabetes: Post Hoc Analyses of the SUSTAIN 6 and PIONEER 6 Trials,” Diabetes, Obesity & Metabolism 25, no. 8 (2023): 2388–2392, 10.1111/dom.15081. 37016488

[44] M. Husain , S. C. Bain , O. K. Jeppesen , et al., “Semaglutide (SUSTAIN and PIONEER) Reduces Cardiovascular Events in Type 2 Diabetes Across Varying Cardiovascular Risk,” Diabetes, Obesity & Metabolism 22, no. 3 (2020): 442–451, 10.1111/dom.13955. PMC7064975 31903692

[45] A. M. Lincoff , K. Brown‐Frandsen , H. M. Colhoun , et al., “Semaglutide and Cardiovascular Outcomes in Obesity Without Diabetes,” New England Journal of Medicine 389, no. 24 (2023): 2221–2232, 10.1056/NEJMoa2307563. 37952131

[46] D. E. Garcia , M. D. Lucas , L. Perez‐Belmonte , et al., “Results of Semaglutide in Patients Older Than 70 Years, a Real‐World Study of Efficacy and Safety,” Minerva Endocrinol (Torino) (2023), 10.23736/S2724-6507.23.03985-4. 37337740

[47] V. Perkovic , K. R. Tuttle , P. Rossing , et al., “Effects of Semaglutide on Chronic Kidney Disease in Patients With Type 2 Diabetes,” New England Journal of Medicine 391, no. 2 (2024): 109–121, 10.1056/NEJMoa2403347. 38785209

[48] M. N. Kosiborod , M. C. Petrie , B. A. Borlaug , et al., “Semaglutide in Patients With Obesity‐Related Heart Failure and Type 2 Diabetes,” New England Journal of Medicine 390, no. 15 (2024): 1394–1407, 10.1056/NEJMoa2313917. 38587233

[49] O. Baser , G. Samayoa , K. Rodchenko , L. Isenman , E. Baser , and N. Yapar , “The Association Between Weight Loss Medications and Cardiovascular Complications,” Obesity (Silver Spring) 32, no. 7 (2024): 1401–1409, 10.1002/oby.24037. 38706431

[50] A. M. Jastreboff , L. J. Aronne , N. N. Ahmad , et al., “Tirzepatide Once Weekly for the Treatment of Obesity,” New England Journal of Medicine 387, no. 3 (2022): 205–216, 10.1056/NEJMoa2206038. 35658024

[51] W. T. Garvey , J. P. Frias , A. M. Jastreboff , et al., “Tirzepatide Once Weekly for the Treatment of Obesity in People With Type 2 Diabetes (SURMOUNT‐2): A Double‐Blind, Randomised, Multicentre, Placebo‐Controlled, Phase 3 Trial,” Lancet 402, no. 10402 (2023): 613–626, 10.1016/S0140-6736(23)01200-X. 37385275

[52] L. J. Aronne , N. Sattar , D. B. Horn , et al., “Continued Treatment With Tirzepatide for Maintenance of Weight Reduction in Adults With Obesity: The SURMOUNT‐4 Randomized Clinical Trial,” JAMA 331, no. 1 (2024): 38–48, 10.1001/jama.2023.24945. 38078870 PMC10714284

[53] M. A. Abdalla , I. Soyiri , S. Atkin , and T. Sathyapalan , “Tirzepatide as a Novel Therapeutic Option for Patients With Type 2 Diabetes: A Pooled Analysis of Subgroups of SURPASS 1–5 Trials,” Journal of Diabetology 14, no. 2 (2023): 65–73, 10.4103/jod.jod_16_23.

[54] A. Kiyosue , J. P. Dunn , X. Cui , et al., “Safety and Efficacy Analyses Across Age and Body Mass Index Subgroups in East Asian Participants With Type 2 Diabetes in the Phase 3 Tirzepatide Studies (SURPASS Programme),” Diabetes, Obesity and Metabolism 25, no. 4 (2023): 1056–1067, 10.1111/dom.14952. 36545807

[55] M. Packer , M. R. Zile , C. M. Kramer , et al., “Tirzepatide for Heart Failure With Preserved Ejection Fraction and Obesity,” New England Journal of Medicine 392, no. 5 (2025): 427–437, 10.1056/NEJMoa2410027. 39555826

[56] A. Malhotra , J. Bednarik , S. Chakladar , et al., “Tirzepatide for the Treatment of Obstructive Sleep Apnea: Rationale, Design, and Sample Baseline Characteristics of the SURMOUNT ‐OSA Phase 3 Trial,” Contemporary Clinical Trials 141 (2024): 107516, 10.1016/j.cct.2024.107516. 38547961 PMC11168245

[57] S. Liabeuf , R. Minutolo , J. Floege , and C. Zoccali , “The Use of SGLT2 Inhibitors and GLP‐1 Receptor Agonists in Older Patients: A Debate on Approaches in CKD and Non‐CKD Populations,” Clinical Kidney Journal 18, no. 2 (2024): sfae380, 10.1093/ckj/sfae380. 39906070 PMC11788569

[58] K. Lian , K. Zhang , C. Kan , et al., “Emerging Therapeutic Landscape: Incretin Agonists in Chronic Kidney Disease Management,” Life Sciences 351 (2024): 122801, 10.1016/j.lfs.2024.122801. 38862060

[59] X. Wang , X. Yang , X. Qi , et al., “Anti‐Atherosclerotic Effect of Incretin Receptor Agonists,” Frontiers in Endocrinology 15 (2024): 1463547, 10.3389/fendo.2024.1463547. 39493783 PMC11527663

[60] S. Nicholls , D. Bhatt , J. Buse , et al., “Comparison of Tirzepatide and Dulaglutide on Major Adverse Cardiovascular Events in Participants With Type 2 Diabetes and Atherosclerotic Cardiovascular Disease: SURPASS‐CVOT Design and Baseline Characteristics,” American Heart Journal 267 (2024): 1–11, 10.1016/j.ahj.2023.09.007. 37758044

[61] “American Geriatrics Society 2023 Updated AGS Beers Criteria® for Potentially Inappropriate Medication Use in Older Adults,” Journal of the American Geriatrics Society 71, no. 7 (2023): 2052–2081. 37139824 10.1111/jgs.18372 PMC12478568

[62] P. N. Newsome , K. Buchholtz , K. Cusi , et al., “A Placebo‐Controlled Trial of Subcutaneous Semaglutide in Nonalcoholic Steatohepatitis,” New England Journal of Medicine 384, no. 12 (2021): 1113–1124, 10.1056/NEJMoa2028395. 33185364

[63] R. Loomba , M. L. Hartman , E. J. Lawitz , et al., “Tirzepatide for Metabolic Dysfunction‐Associated Steatohepatitis With Liver Fibrosis,” New England Journal of Medicine 391, no. 4 (2024): 299–310, 10.1056/NEJMoa2401943. 38856224

[64] L. Wang , R. Xu , D. C. Kaelber , and N. A. Berger , “Glucagon‐Like Peptide 1 Receptor Agonists and 13 Obesity‐Associated Cancers in Patients With Type 2 Diabetes,” JAMA Network Open 7, no. 7 (2024): e2421305, 10.1001/jamanetworkopen.2024.21305. 38967919 PMC11227080

[65] L. Wang , Q. Wang , L. Li , D. C. Kaelber , and R. Xu , “Glucagon‐Like Peptide‐1 Receptor Agonists and Pancreatic Cancer Risk: Target Trial Emulation Using Real‐World Data,” Journal of the National Cancer Institute 117 (2024): 476–485, 10.1093/jnci/djae260. PMC11884861 39418202

[66] S. Li , X. Xiao , and X. Zhang , “Hydration Status in Older Adults: Current Knowledge and Future Challenges,” Nutrients 15, no. 11 (2023): 2609, 10.3390/nu15112609. 37299572 PMC10255140

[67] T. Wang , B. Buse , and V. Pate , “Liraglutide as a Potential Drug Repurposing Candidate for Alzheimer's Disease and Related Dementia: Real World Evidence,” Alzheimer's & Dementia 19 (2023): e073346, 10.1002/alz.073346.

[68] C. M. Prado , J. A. Batsis , L. M. Donini , M. C. Gonzalez , and M. Siervo , “Sarcopenic Obesity in Older Adults: A Clinical Overview,” Nature Reviews Endocrinology 20, no. 5 (2024): 261–277, 10.1038/s41574-023-00943-z. PMC12854800 38321142

[69] H. J. Pownall , G. A. Bray , L. E. Wagenknecht , et al., “Changes in Body Composition Over 8 Years in a Randomized Trial of a Lifestyle Intervention: The Look AHEAD Study,” Obesity (Silver Spring) 23, no. 3 (2015): 565–572, 10.1002/oby.21005. 25707379 PMC4707962

[70] D. T. Villareal , L. Aguirre , A. B. Gurney , et al., “Aerobic or Resistance Exercise, or Both, in Dieting Obese Older Adults,” New England Journal of Medicine 376, no. 20 (2017): 1943–1955, 10.1056/NEJMoa1616338. 28514618 PMC5552187

[71] K. M. Beavers , M. F. Lyles , C. C. Davis , X. Wang , D. P. Beavers , and B. J. Nicklas , “Is Lost Lean Mass From Intentional Weight Loss Recovered During Weight Regain in Postmenopausal Women?,” American Journal of Clinical Nutrition 94, no. 3 (2011): 767–774, 10.3945/ajcn.110.004895. 21795437 PMC3155932

[72] J. A. Batsis , D. Gilbert‐Diamond , A. C. McClure , et al., “Prevalence of Sarcopenia Obesity in Patients Treated at a Rural, Multidisciplinary Weight and Wellness Center,” Clinical Medicine Insights. Arthritis and Musculoskeletal Disorders 12 (2019): 1179544119862288, 10.1177/1179544119862288. 31384133 PMC6651666

[73] S. Perna , D. Guido , C. Bologna , et al., “Liraglutide and Obesity in Elderly: Efficacy in Fat Loss and Safety in Order to Prevent Sarcopenia. A Perspective Case Series Study,” Aging Clinical and Experimental Research 28, no. 6 (2016): 1251–1257, 10.1007/s40520-015-0525-y. 26749118

[74] J. P. H. Wilding , R. L. Batterham , S. Calanna , et al., “Once‐Weekly Semaglutide in Adults With Overweight or Obesity,” New England Journal of Medicine 384, no. 11 (2021): 989–1002, 10.1056/NEJMoa2032183. 33567185

[75] T. Heise , J. H. DeVries , S. Urva , et al., “Tirzepatide Reduces Appetite, Energy Intake, and Fat Mass in People With Type 2 Diabetes,” Diabetes Care 46, no. 5 (2023): 998–1004, 10.2337/dc22-1710. 36857477 PMC10154650

[76] M. Look , J. P. Dunn , R. F. Kushner , et al., “Body Composition Changes During Weight Reduction With Tirzepatide in the SURMOUNT‐1 Study of Adults With Obesity or Overweight,” Diabetes, Obesity & Metabolism 27, no. 5 (2025): 2720–2729, 10.1111/dom.16275. PMC11965027 39996356

[77] D. Rubino , J. B. Bjorner , N. Rathor , et al., “Effect of Semaglutide 2.4 Mg on Physical Functioning and Weight‐ and Health‐Related Quality of Life in Adults With Overweight or Obesity: Patient‐Reported Outcomes From the STEP 1–4 Trials,” Diabetes, Obesity and Metabolism 26, no. 7 (2024): 2945–2955, 10.1111/dom.15620. 38698650

[78] J. C. Seidell and T. L. Visscher , “Body Weight and Weight Change and Their Health Implications for the Elderly,” European Journal of Clinical Nutrition 54, no. S3 (2000): S33–S39, 10.1038/sj.ejcn.1601023. 11041073

[79] Y. S. Zhang , W. Y. Weng , B. C. Xie , et al., “Glucagon‐Like Peptide‐1 Receptor Agonists and Fracture Risk: A Network Meta‐Analysis of Randomized Clinical Trials,” Osteoporosis International 29, no. 12 (2018): 2639–2644, 10.1007/s00198-018-4649-8. 30083774

[80] Y. Zhang , G. Chen , W. Wang , D. Yang , D. Zhu , and Y. Jing , “Association of Glucagon‐Like Peptide‐1 Receptor Agonists Use With Fracture Risk in Type 2 Diabetes: A Meta‐Analysis of Randomized Controlled Trials,” Bone 192 (2025): 117338, 10.1016/j.bone.2024.117338. 39603373

[81] J. R. Lundgren , C. Janus , S. B. K. Jensen , et al., “Healthy Weight Loss Maintenance With Exercise, Liraglutide, or Both Combined,” New England Journal of Medicine 384, no. 18 (2021): 1719–1730, 10.1056/NEJMoa2028198. 33951361

[82] J. Rosenstock , C. Wysham , J. P. Frías , et al., “Efficacy and Safety of a Novel Dual GIP and GLP‐1 Receptor Agonist Tirzepatide in Patients With Type 2 Diabetes (SURPASS‐1): A Double‐Blind, Randomised, Phase 3 Trial,” Lancet 398, no. 10295 (2021): 143–155, 10.1016/S0140-6736(21)01324-6. 34186022

[83] K. Windahl , N. C. Chesnaye , G. F. Irving , et al., “The Safety of a Low‐Protein Diet in Older Adults With Advanced Chronic Kidney Disease,” Nephrology, Dialysis, Transplantation 39, no. 11 (2024): 1867–1875, 10.1093/ndt/gfae077. PMC11648958 38544335

[84] K. Kalantar‐Zadeh , H. M. Kramer , and D. Fouque , “High‐Protein Diet Is Bad for Kidney Health: Unleashing the Taboo,” Nephrology, Dialysis, Transplantation 35, no. 1 (2020): 1–4, 10.1093/ndt/gfz216. 31697325

[85] “Calcium Fact Sheet for Health Professionals,” National Institutes of Health Office of Dietary Supplements, last modified July 11, 2025, https://ods.od.nih.gov/factsheets/Calcium‐HealthProfessional/

[86] C. J. Haywood , L. A. Prendergast , R. Lim , M. Lappas , W. K. Lim , and J. Proietto , “Obesity in Older Adults: Effect of Degree of Weight Loss on Cardiovascular Markers and Medications,” Clinical Obesity 9, no. 4 (2019): e12316, 10.1111/cob.12316. 31207126

